# Direct‐acting antiviral therapy for chronic hepatitis C virus genotype 4 infection: Exploring new regimens

**DOI:** 10.1002/hsr2.106

**Published:** 2019-03-04

**Authors:** Hidenori Toyoda

**Affiliations:** ^1^ Department of Gastroenterology Ogaki Municipal Hospital Ogaki Japan

## Abstract

The optimal antiviral regimen for hepatitis C virus (HCV) genotype 4 is still a subject of investigation. The report in this issue of *Health Science Reports* by Asselah and colleagues investigated the additional benefit of extending treatment duration of the ombitasvir/paritaprevir/ritonavir plus ribavirin regimen to up to 24 weeks for patients with HCV genotype 4 and compensated cirrhosis.

## Abstract 2

This article discusses: Ombitasvir/paritaprevir/ritonavir plus ribavirin for 24 weeks in patients with HCV GT4 and compensated cirrhosis (AGATE‐I Part II)

1

HCV genotype 4 accounts for over 8% of all patients with HCV infection globally. This genotype is predominantly found in patients from Egypt. Its prevalence varies widely by regions, with the highest prevalence reported in the Middle East and sub‐Saharan Africa.^1^ The geographic distribution of patients with HCV genotype 4 infection is increasing, however, due to migration.

Four all‐oral direct‐acting antiviral (DAA) combination therapy regimens are recommended as anti‐HCV therapy for treatment‐naïve patients with HCV genotype 4 infection in the guidelines from the European Association for the Study of the Liver (EASL)^2^ and the American Association for the Study of Liver Diseases (AASLD)–Infectious Diseases Society of America (IDSA)^3^: sofosbuvir/velpatasvir, glecaprevir/pibrentasvir, sofosbuvir/ledipasvir, and grazoprevir/elbasvir. For treatment‐experienced patients, on the other hand, the EASL guidelines recommend sofosbuvir/velpatasvir or glecaprevir/pibrentasvir, and the AASLD–IDSA guidelines recommend only the sofosbuvir/velpatasvir/voxilaprevir regimen. All of these regimens have shown high virologic efficacy against genotype 4, with sustained virologic response rates ranging 91%‐100%^4^. While these recommendations are based on several clinical trials that have included patients with HCV genotype 4, in most of these trials, patients with HCV genotype 4 were grouped with patients with HCV genotype 1 or several other genotypes. In addition, patients with HCV genotype 4 were in the minority in these trials, probably because of the small number of patients with HCV genotype 4 in the locations where these trials were conducted; indeed, most trials included less than 100 patients with HCV genotype 4. Thus, the optimal regimen for HCV genotype 4, including the best DAAs and treatment duration, is still a subject of investigation. The report in this issue of *Health Science Reports* by Asselah et al^5^ investigated the additional benefit of extending treatment duration of the ombitasvir/paritaprevir/ritonavir plus ribavirin regimen, which was not included in the current treatment guidelines for HCV genotype 4, to up to 24 weeks for patients with HCV genotype 4 and compensated cirrhosis. This study is Part II of AGATE‐I trial,^6^ the multinational open‐label trial for the evaluation of the efficacy, tolerability, and safety of this regimen in patients with genotype 4. AGATE‐I Part I trial included two Arms: Arm A, with patients treated for 12 weeks, and Arm B, with patients treated for 16 weeks. In Part II, reported in this issue,^5^ two additional Arms were evaluated: Arm C, which included treatment‐naïve patients or patients with a history of treatment with interferon/peginterferon plus ribavirin, and Arm D, which included patients with a history of sofosbuvir/peginterferon plus ribavirin or sofosbuvir plus ribavirin. Patients were treated for 24 weeks in both Arms. The efficacy, tolerability, and safety were comparable across all four Arms. The extension of treatment duration to 24 weeks did not show superior efficacy to 12 or 16 weeks. In addition, longer treatment duration did not offer additional benefit on short‐term regression of liver fibrosis. These negative results are important for establishing the optimal regimen for patients with HCV genotype 4.

There is substantial genetic heterogeneity in HCV genotype 4, and many subgenotypes have been reported, although, with the exception of subtypes 4a and 4b, the prevalence of these subtypes is low. The efficacy of DAA regimens by subgenotype is unclear and will be the subject of further research. Although some trials that focused on patients with HCV genotype 4 have included more than 100 patients,^6^, ^7^ most patients had HCV subgenotype 4a or 4d. The most recent study by Fourati et al^8^ reported an unexpectedly high rate of treatment failure in patients with HCV subgenotype 4r, a genotype which has been reported in African countries but that is rarely found in regions such as Europe and North America, which is associated with a high frequency of baseline resistance‐associated substitutions. Based on their report, it could be proposed that subgenotype evaluation should be recommended in patients with HCV genotype 4 when selecting a treatment regimen. However, given the low proportion of 4r in the overall HCV‐infected population and the lack of available commercial assays to identify HCV genotype 4 subtypes,^9^ universal subtyping of all genotype 4‐infected patients to select regimen may be difficult to implement in actual clinical practice at this time.

The ideal DAA regimen for HCV genotype 4 has not been fully established, although there are multiple DAA treatment options for patients with HCV genotype 4, particularly those with the predominant subtypes, and overall efficacy in clinical trials has been reported to be as high as with other genotypes. We expect the accumulation of more findings on the efficacy of various regimens, and on different subtypes, perhaps based on real‐world results, to confirm the high rates observed of sustained virologic response in patients with HCV genotype 4.

## CONFLICTS OF INTEREST

None declared.
